# PCA Based Stress Monitoring of Cylindrical Specimens Using PZTs and Guided Waves

**DOI:** 10.3390/s17122788

**Published:** 2017-12-01

**Authors:** Jabid Quiroga, Luis Mujica, Rodolfo Villamizar, Magda Ruiz, Johanatan Camacho

**Affiliations:** 1 Schools of Mechanical and Electric, Electronics and Telecommunications Engineering, Universidad Industrial de Santander (UIS), Cra 27 Calle 9, 680002 Bucaramanga, Colombia; rovillam@uis.edu.co; 2 Departament de Matemàtiques, CoDAlab, Escola d’Enginyeria de Barcelona Est (EEBE), Universitat Politècnica de Catalunya, Campus Diagonal-Besòs. C, Eduard Maristany, 6-12, St. Adrià de Besòs, 08930 Barcelona, Spain; luis.eduardo.mujica@upc.edu (L.M.); magda.ruiz@upc.edu (M.R.); camacho.navarro.jhonatan@gmail.com (J.C.)

**Keywords:** acoustoelasticity, stress monitoring, guided waves, piezoelectrics, statistical analysis, cylindrical waveguide

## Abstract

Since mechanical stress in structures affects issues such as strength, expected operational life and dimensional stability, a continuous stress monitoring scheme is necessary for a complete integrity assessment. Consequently, this paper proposes a stress monitoring scheme for cylindrical specimens, which are widely used in structures such as pipelines, wind turbines or bridges. The approach consists of tracking guided wave variations due to load changes, by comparing wave statistical patterns via Principal Component Analysis (PCA). Each load scenario is projected to the PCA space by means of a baseline model and represented using the *Q*-statistical indices. Experimental validation of the proposed methodology is conducted on two specimens: (i) a 12.7 mm ( 1/2″ ) diameter, 0.4 m length, AISI 1020 steel rod, and (ii) a 25.4 mm ( 1″ ) diameter, 6m length, schedule 40, A-106, hollow cylinder. Specimen 1 was subjected to axial loads, meanwhile specimen 2 to flexion. In both cases, simultaneous longitudinal and flexural guided waves were generated via piezoelectric devices (PZTs) in a pitch-catch configuration. Experimental results show the feasibility of the approach and its potential use as in-situ continuous stress monitoring application.

## 1. Introduction

Many structures are exposed to load changes caused by environmental and operation conditions such as temperature and external force variations, which produce high stress variations. This condition can affect the system integrity and a catastrophic condition could be present. Thus, for some cases it is imperative to continuously trace variations of the stress condition, where the most widely used transducer is the strain gage with limitations such as: (i) the high influence of the adhesive layer on its performance; (ii) its focus on stress measurement in the coupling point; and (iii) that prestresses are not exposed once the sensor is attached to the specimen. On the other hand, stress measurement based on the Acoustoelastic effect (stress dependence of ultrasonic bulk velocity) is a low cost method that has recently been used in several industrial applications. However, this method is slightly sensitive to the microstructure effects like grain size [1], texture and structure [2,3] and operational conditions such as temperature variations [4] and sensor coupling [5]. Ultrasonic guided waves, unlike bulk waves and other available NDT methods, have the ability of propagating along relative long distances over the waveguide, preserving its sensitivity to structural condition changes. Thus, guided waves are used for damage detection, damage localization and material characterization.

When guided waves are compared for different stress states, phase shifts are mainly attributed to traveling distance changes of the guided wave (actuator-sensor) and to velocity changes due to the acoustoelasticity effect. Based on the previous phenomena, estimation of stress in a waveguide could be achieved by measuring the Time Of Flight (TOF) of the guided wave among piezoelectric transducers. However, this is a complex task given: (i) TOF changes are small in the stressed specimen or waveguide. For example, for metals, TOF variation is less than 0.001 per MPA of applied stress [6], which demands high precision in the experimentation; (ii) the presence of dispersion and multimode propagation and (iii) wavefront overlapping due to the waveguide shape when a circular propagation is generated. Additionally, bulk propagation velocities due to the acuoelasticity phenomenon also depend on the inherent or induced preferred orientation of crystalline grains and the localized plastic deformation (closely related to residual stresses) [3].

Based on the above, the multivariable nature of the affectation of the wave pattern when the guided wave is exposed to a propagation under a stress medium is evident. Thus, a multivariable statistical tool such as Principal Component Analysis (PCA) can be used as a data-driven modeling approach offering a suitable alternative to track changes in the guided wave produced by the stress. PCA has been previously applied for extracting structural damage features [7]; detection of nonlinearity effects in structural integrity monitoring of offshore jacket-type structures [8], and a simplified solar array system [9]; dimensionality reduction of multiple sensor arrays in structure damage classification [10]; evaluating progressive cracks in a steel sheet and turbine blade of an aircraft [11]; evaluating the tensile stress in a rod [12]; and distinguishing abnormal condition [13].

In general, this paper considers a methodology to trace variations on stress in the waveguide representing the guided wave pattern into PCA statistical space. This paper is organized as follows. A brief reviewing of ultrasonic stress monitoring techniques is presented in Section 2, Section 3 is focused on the description of the theoretical background of the acoustoelasticity effect and Principal Component Analysis. The proposed stress monitoring methodology and novelty of this paper is explained in details in Section 4. In Section 5, a description of the test benches used to demonstrate the proposal is defined. Results are depicted in Section 6. Finally, conclusions and the main contributions of this paper are drawn in Section 7.

## 2. Review of Ultrasonic Stress Monitoring Techniques

Most of the works on ultrasonic stress monitoring, reported in literature, are focused on measuring the Time Of Flight (TOF) or phase shift to determine phase and group velocities and comparing them with a knowledge base. Di Scalea and Rizzo [14] present a method based on guided waves for stress monitoring in seven-wire steel strands, where discrepancies in the group velocity on single (straight) wires with respect to the theoretical predictions, at low stress levels are observed. It is also found that the use of low excitation frequencies would help to minimize the influence of the individual wires on the wave propagation behavior in the multiwire strands. Additionally, a substantial increase in stress measurement sensitivity is accomplished by adding the effect of strand elongation. Chaki et al., in [15,16] report the use of the relative phase velocity change of the Longitudinal L(0, 1) mode for estimating the stress levels in a prestressed steel strands by means of an analytic model and an acoustoelastic calibration curve. The analytical model depends on the material properties, waveguide diameter and probing frequency. In [17], the authors studied the stress in bolted connections by comparing several methods based on the acoustoelasticity effect (TOF, velocity ratio and mechanical resonance frequency shift). They found small velocity changes in presence of stress variation, which demands a high sampling rate and consequently an increased cost. This can be associated with several factors, such as the material microstructure, environmental noise and bonding layer thickness, which can affect the measurement accuracy of TOF. In addition, it is observed that changes in the bolt preload influence the dynamic characteristics of the interface, affecting the propagation of the ultrasonic wave through the interface. Therefore, by analyzing the received ultrasonic signal, the bolt connection condition can be estimated. In [18] the homogeneous biaxial stresses are assessed for an aluminum plate by measuring phase velocity changes on multiple propagation directions using a single mode at a specific frequency. Experimental results indicate that phase velocity changes can be closely approximated by a sinusoidal function with respect to propagation angle, where every sinusoidal coefficient can be estimated with a single uniaxial loading experiment. However, this approach is not practical since a great experimental effort is required to define the sinusoidal function and it is highly sensitive to the material microstructure. Finally, in [19], the authors used the SAFE method to analyze in a rail the influence of axial load on the wave propagation up to 100 kHz. Although it is possible to measure the phase shift caused by axial load changes, the sensitivity to changes in elastic modulus due to temperature changes is of a larger magnitude. They concluded the necessity of compensating or eliminating external effects, mainly propagation changes due to temperature variations, in order to achieve a robust estimation of the stress.

## 3. Theoretical Framework

### 3.1. Acoustoelasticity Effect

Murnaghan proposed in [20] the theory of finite deformation on the bulk elastic waves propagation for an initially isotropic solid subjected to a stress. Then, Hughes and Kelly, based on this work, developed the theoretical framework that explains the dependence of bulk wave speed under stress, or the acoustoelasticity effect [21]. They specifically considered uniaxial stress and derived expressions for changes in shear and longitudinal bulk wave velocities as a function of the applied stress for known material properties. It was determined that for isotropic materials subjected to uniaxial stress, three constants *l, m, n* are required to describe the relation between stress and velocity, in addition to the two Lamé constants, λ and μ . Once the *l, m, n* values of a particular material specimen are determined, experimental measurements of one of the bulk velocities reveals the stress level that the specimen is subjected to. For the case of a homogeneous and isotropic infinite solid subjected to a uniaxial stress, the bulk velocities of longitudinal $C_{1}$ and shear waves $C_{2}$ , propagating in the same direction as the applied stress, can be written in the first-order approximation of Equations (1) and (2), respectively, as [16]:

(1) $$C_{1}^{\sigma }=\sqrt{\frac{\lambda +2\mu }{\rho }}\left\{1+\frac{\sigma }{2(\lambda +2\mu )(2\lambda +2\mu )}\left(\frac{\lambda +\mu }{\mu }\left(4\lambda +10\mu +4m\right)+\lambda +2l\right)\right\}$$

(2) $$C_{2}^{\sigma }=\sqrt{\frac{\lambda }{\rho }\left\{1+\frac{\sigma }{2\mu (3\lambda +2\mu )}\left(4\lambda +4\mu +m+\frac{\lambda n}{4\mu }\right)\right\}}$$

where ρ is the mass density, σ is the tensile stress, and *m*, *n* and *l* are the Murnaghan’s Third Order Elastic Constants (TOEC). Expressions inside curlic brackets represent the effect of the stress on the bulk velocity (acuostoelastic effect).

The analytic approach for computing dispersion curves of guided waves propagating along the axial direction of a cylindrical waveguide is based on the uncoupled wave Equations (3) and (4)

(3) $$C_{1}\nabla^{2}\Phi =\frac{\partial^{2}\Phi }{\partial t^{2}}$$

(4) $$C_{2}\nabla^{2}H=\frac{\partial^{2}H}{\partial t^{2}}$$

where $C_{1}$ and $C_{2}$ , are the longitudinal and shear bulk velocities, respectively, and Φ and *H* are the scalar and vectorial field potentials, respectively. According to Equations (1) and (2), it is observed that bulk velocities $C_{1}$ and $C_{2}$ are stress dependent, which are used to compute the phase ( $V_{p}$ ) and group ( $V_{g}$ ) guided wave velocities. These velocities, phase and group, are frequency dependent and they are typically depicted in the dispersion curves. Such curves are computed for unstressed samples based on the eigenvalues of the stress equation with zero stress at the boundaries. As it can be inferred and presented in several works, e.g., [19,22,23,24,25], the presence of stress in the propagation path yields variation in the velocity of the guided waves compared with the free-stress case. So, it can be concluded that phase and group velocities in cylindrical waveguides depend not only on frequency but also on applied stress $V_{p}=f_{p}\left(\omega ,\sigma \right)$ and $V_{g}=f_{g}\left(\omega ,\sigma \right)$ . Although, the acoustoelasticity effect is applied to determine the ultrasonic bulk velocities, some recent experimental works have shown that guided wave velocities magnitude are affected by stress. Slight changes of this effect are reported by [15,16], where the longitudinal mode L(0, 1) is used to track variations of the phase velocity in a waveguide rod-like subjected to stress. In addition, theoretical approaches have been recently proposed to determine the dispersion curves for specimens under stress. In the case of lamb waves, Gandhi et al., [25] determine the dispersion curves for plates based on the acoustoelasticity theory and, for the case of prismatic waveguides, Loveday et al., [23] present an approach based on a Semianalytical Finite Element (SAFE) frame.

### 3.2. Principal Components Analysis (PCA)

PCA is used in this approach as a data driven modeling technique in order to represent the wave pattern at different structural stress conditions in a new reduced space. This statistical tool has been extensively applied for extracting structural damage features and to discriminate features from damaged and undamaged structures [11,13,26].

The main objective of PCA is to distinguish between the most relevant dynamics changes of the system, the redundant information and signal noise. This objective is essentially accomplished by defining a new coordinate space where the variance is maximized and correlation between variables is minimized. In other words, the objective is to find a linear transformation orthogonal matrix $P\in M_{I\times K}\left(R\right)$ for transforming the original data matrix X into the form:

(5) $$T=\mathrm{XP}\in M_{I\times K}\left(R\right).$$

where the matrix P contains the principal components of the data set or loading matrix and the matrix T is the transformed or projected matrix onto the principal component space, also called scores matrix. The columns of P are the eigenvectors of the covariance matrix $C_{X}$ organized in descending order of its associated eigenvalue. In this way,

(6) $$C_{X}P=P\Lambda ,$$

where

(7) $$C_{X}=\frac{1}{K-1}X^{T}X\in M_{I\times K}\left(R\right),$$

and Λ is a diagonal matrix that contains the eigenvalues $\lambda_{i},\;i=1,2,\ldots ,K$ .

The eigenvector with the highest eigenvalue is the most representative component for the data with the largest quantity of information. Geometrically, the *j*th-column vector ( $t_{j}$ ) of the transformed data matrix **T** is the projection of the original data over the direction of vector $p_{j}$ (*j*th principal component). The projected data in the new space are uncorrelated and have maximal variance, thus it can be potentially the best representation of the process features. Since eigenvectors are ordered according to variance, it is possible to reduce the dimensionality of the data set **X** by choosing only a reduced number, ϱ<K , of eigenvectors related to the ϱ highest eigenvalues. In this way, given the reduced matrix $\hat{P}\in M_{K\times \varrho }\left(R\right)$ , the score matrix is defined as

(8) $$\hat{T}=X\hat{P}\in M_{I\times \varrho }\left(R\right).$$

$\hat{T}$ can be projected back onto the original *K*-dimensional space to obtain a reconstructed data matrix as follows:

(9) $$\hat{X}=\hat{T}{\hat{P}}^{T}\in M_{I\times \cdot K}\left(R\right).$$

The difference between the original matrix X and the reconstructed one $\hat{X}$ describes the unrepresented variability in the projections and it is defined as the residual error matrix E as follows:

$$\begin{array}{cc} E & =X-\hat{X}\\ & =X-\hat{T}{\hat{P}}^{T}\\ & =X-X\hat{P}{\hat{P}}^{T}, \end{array}$$

so

(10) $$\begin{array}{cc} E & =X\left(I-\hat{P}{\hat{P}}^{T}\right)\in M_{I\times K}\left(R\right) \end{array}$$

For simplicity, the caret is removed from the reduced matrices ( $\hat{T}$ and $\hat{P}$ ) in the rest of the paper. If Equations (8) and (10) are analyzed in terms of experimental trials, it can be obtained that:

(11) $$t_{i}^{T}=x_{i}^{T}P\in R^{\varrho },\;i=1,\ldots ,I$$

and

(12) $$e_{i}^{T}=x_{i}^{T}\left(I-PP^{T}\right)\in R^{K},\;i=1,\ldots ,I$$

where, $x_{i}^{T}\in R^{K}$ denotes the vector of the *i*th experimental trial (*i*th row of the original matrix X ), vector $t_{i}^{T}$ is the projection of $x_{i}$ onto the first ϱ principal components (*i*th row of the score matrix T ) and $e_{i}^{T}$ is the residual error of the *i*th experimental trial (*i*th row of matrix E ).

- PCA Based index
  One well-known PCA statistical index used to distinguish abnormal behavior in a process is the *Q*-statistic or Square Prediction Error (SPE)-statistic.This index uses the residual error matrix E to represent the variability of the data projected on the residual subspace. The *Q*-statistic is based on the assumption that the underlying process follows approximately a multivariate normal distribution, where the first moment vector is zero. Therefore, this index denotes that events are unexplained by the reduced model. In other words, it is a measurement of the difference, or residual, between a sample and its retrieved version by using the reduced model. The *Q*-statistic of the *i*th experimental trial is defined as the sum of the squared residuals of each variable as follows:
(13)Qi=∥ei∥2=eiTei=∑ℓ=1Kei,ℓ2(14)=xiTI−PPTI−PPTTxi(15)=xiTI−PPT−PPT+PPTxi(16)=xiTI−PPTxi
  where $e_{i,\ell }\in R$ denotes the *ℓ*th element of the vector $e_{i}$ , ℓ=1,…,K .

## 4. PCA Based Stress Monitoring Approach

**Figure 1:**
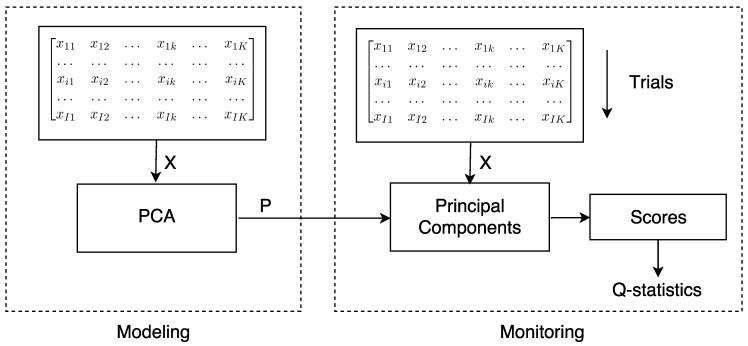
General scheme of the proposed PCA based stress monitoring.

This section presents the contribution of this work in the field of stress monitoring in cylindrical structures, experimentally validated in a stressed and piezo-actuated steel rod and tube. The proposed methodology for stress monitoring based on PCA consists of two stages: (i) modeling and (ii) stress monitoring (see Figure 1).

### 4.1. Modeling

In this stage, a statistical data driven model for the nominal condition of the piezo-actuated specimen is constructed by using PCA. The next steps are followed:

1. A set of *I* experiments are conducted on the specimen at nominal condition (residual or initial stress). The experiment consists of exciting the specimen by a PZT, via a modulated pulse at a single probe position and capturing the guided wave by a PZT, at a point distant from the excitation, such that the interest zone is covered. This measurement is repeated several times (experimental trials). The collected data are arranged as follows:
(17) $$X=\left[\begin{array}{cccccc} x_{11} & x_{12} & \cdots & x_{1k} & \cdots & x_{1K}\\ \cdots & \cdots & \cdots & \cdots & \cdots & \cdots \\ x_{i1} & x_{i2} & \cdots & x_{ik} & \cdots & x_{iK}\\ \cdots & \cdots & \cdots & \cdots & \cdots & \cdots \\ x_{I1} & x_{I2} & \cdots & x_{Ik} & \cdots & x_{IK} \end{array}\right]=\left[\begin{array}{c} x_{1}\\ \cdots \\ x_{i}\\ \cdots \\ x_{I} \end{array}\right]=(v_{1}|v_{2}\left|\cdots \right|v_{k}\left|\cdots \right|v_{k})$$
  This $X\in M_{I\times K}\left(R\right)$ is the vector space of I×K matrices over R , which contains information from *K* discretization instant times and *I* experimental trials. Each row vector $\left(x_{i}\right)$ represents measurements from the sensor at a specific *i*th trial. In the same way, each column vector $\left(v_{k}\right)$ represents measurements at the specific *k*th discretization instant time in the whole set of experiments trials.
2. Cross correlation analysis is applied between the acting and sensing signals of the *I* experiments to eliminate noisy data trends.The cross-correlation function between two signals *X*(*t*) and *Y*(*t*) is defined by Equation (18).
(18) $$R_{XY}\left(t,t+\tau \right)=\lim_{k\to \infty }\frac{1}{K}\sum_{k=1}^{K}X_{k}\left(t\right)Y_{k}\left(t+\tau \right)$$
  where *K* is the number of samples and τ is the lag time interval used to compute the cross-correlation function.
3. The correlated signals are arranged in the matrix $\tilde{X}$ for *I* experiments of *2K-1* samples, conducted on the same scenario in order to consider noise and variance due to the stochastic nature of the technique.
4. The matrix $\tilde{X}$ is normalized by considering each column as a measured variable and normalized to mean zero and variance equal to one for the *I* experiments. This step minimizes bias and scale variance effects. The Equations (19)–(21) are used for the mentioned preprocessing.
(19) $$\mu_{j}=\frac{1}{I}\sum_{i=1}^{I}x_{ij},j=1,\ldots ,\left(2K-1\right)$$
(20) $$\mu =\frac{1}{I(2K-1)}\sum_{i=1}^{I}\sum_{j=1}^{2K-1}x_{ij}$$
(21) $$\sigma =\sqrt{\frac{1}{I(2K-1)}\sum_{i=1}^{I}\sum_{j=1}^{\left(2K-1\right)}{\left(x_{ij}-\mu \right)}^{2}}$$
  where $\mu_{j}$ is the mean of the experimental trials in the same column of $\tilde{X}$ , and μ and σ are the mean and standard deviation of all elements of $\tilde{X}$ .

Thus, a normalized cross-correlated undamaged matrix $\tilde{x}$ is obtained in a new reduced space of coordinates with minimal redundancy and whose elements correspond to the scaled values of the raw data according to:

(22) $${\tilde{x}}_{ij}=\frac{x_{ij}-\mu_{j}}{\sigma },i=1,\ldots ,I,j=1,\ldots ,\left(2K-1\right)$$

Then, a baseline statistical model is obtained, which consists of a linear transformation (**P**) extracted from the singular value decomposition of the covariance matrix $\tilde{x}$ , according to that described in the previous section.

### 4.2. Monitoring

The monitoring stage consists of representing the currently acquired signals into the PCA space by means of the *Q-Statistics* using Equation (8). The monitoring principle consists of relating changes in the guided wave produced by the presence of stress with changes in the *Q-Statistics* index. In this phase, several experiments are conducted over the steel rod and the tube under load conditions, in order to discriminate different stress states by computing the *Q-Statistics* index and comparing it with the baseline values. The general procedure for detecting and distinguishing stresses on structures based on PCA is depicted in Figure 1.

## 5. Experimental Setup

In order to cover a wide range of stress distributions acting in cylindrical waveguides, experimental tests were conducted on two different steel specimens (solid and hollow cylinder); each one is subjected to different stress profiles over the cross-section of the waveguide. For the case of the rod (solid cylinder), the axial stress is applied perpendicular to the cross-section producing an uniform stress distribution. In the case of the pipe (hollow cylinder), the generated normal stress distribution produced by the pipe bending will vary linearly with the radius, yielding compression and tension stresses in the same cross-section.

### 5.1. Steel Rod

**Figure 2:**
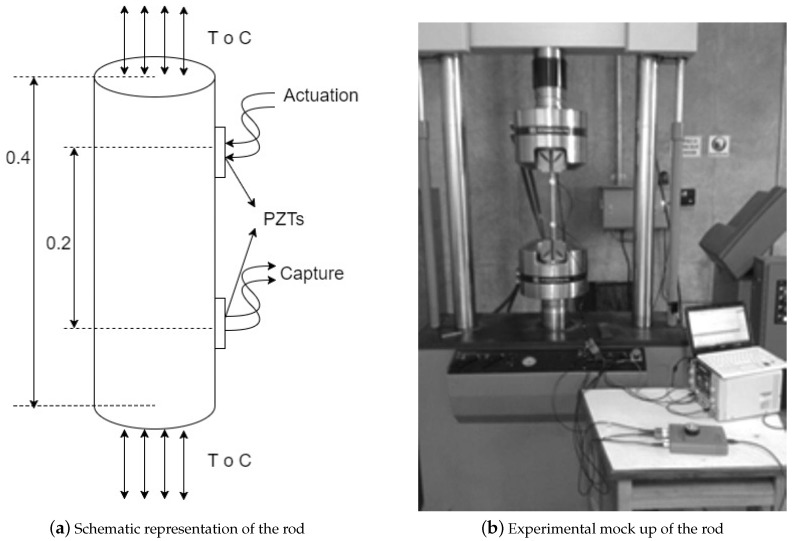
Rod test bench.

A 12.7 mm ( 1/2″ ) diameter, 0.4m length, AISI 1020 steel rod ( E=200 GPa and μ=0.29 ) is instrumented with two PZT’s, separated by 0.4 m, in a pitch-catch configuration, (see Figure 2a). The specimen is subjected to stress by a servohydraulic, MTS universal machine mod 810 (see Figure 2b). The stress nominal condition is determined by the unload condition while stressed scenarios are setup with tension and compression axial loads increasing in magnitude from 5 to 10 kN in steps of 1 kN, where each step belongs to a different stress scenario, named S1, S2, and so on consecutively until S6 (25% of the yield stress). The studied stresses in the specimen are in the lower part of the stress-strain diagram, which usually is the expected work zone for an element in a structure.

### 5.2. Hollow Cylinder

**Figure 3:**
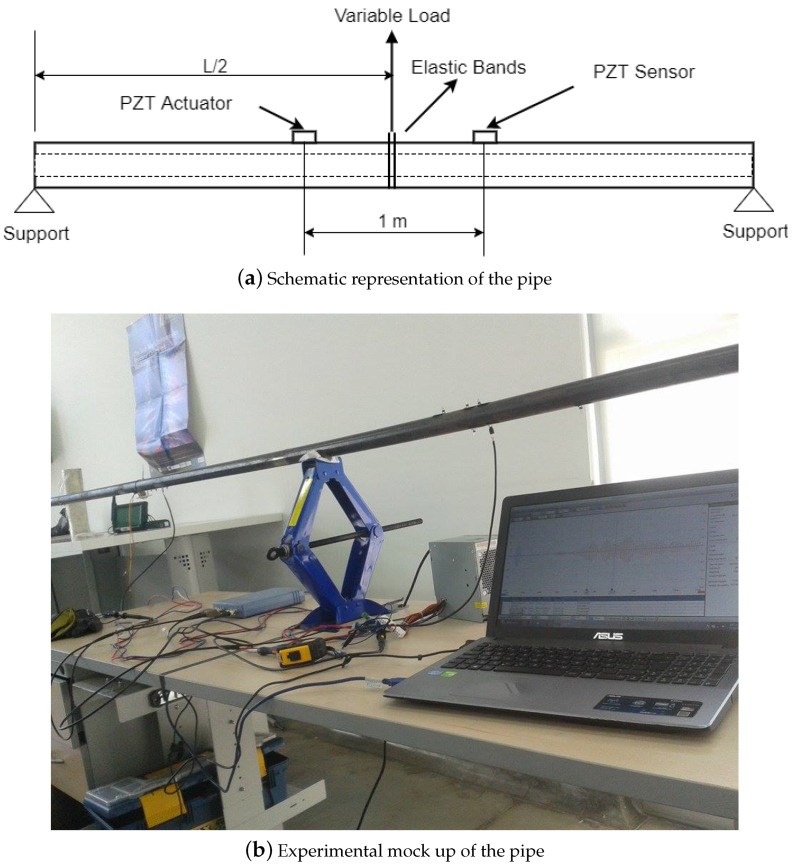
Pipe test bench.

A 25.4 mm ( 1″ ) diameter, 6m length, schedule 40, A-106 ( E=210 GPa and μ=0.33 ), hollow cylinder is supported at the free ends by fixed supports. In this case, it is decided to applied flexion loads to the specimen. The different stress conditions are produced by changing the magnitude of the load in the middle length of the cylinder, $\frac{L}{2}$ , as shown in the Figure 3a. The nominal condition is determined by considering the absence of deflection in the middle length $\frac{L}{2}$ of the cylinder. Under this condition the cylinder is experimenting a negative bending moment and develops an internal stress of 5.96% of the yield strength. Now, the magnitude of the load is changing while the pipeline deflection is increasing in steps of 0.01 m up or down of the original axis position (baseline). Every 0.01 m of deflection constitutes a different stress scenario, in total seven scenarios are studied: five concave upwards deflections and two concave downwards. Concave up deflections are denominated $D_{1}$ for 0.01 m, $D_{2}$ for 0.02 m and so on consecutively until $D_{5}$ for 0.05 m (21.5% of the yield strength), concave down deflections are denominated $D_{6}$ for 0.01 m and $D_{7}$ for 0.02m up of the baseline. Changes in the load magnitude at the $\frac{L}{2}$ position in the cylinder produce axial stresses. So, a simplified but sufficient analytical model is used to track variations in the normal stress because of force variations. Under this scenario the cylinder can be treated as a beam with constant cross-sectional area in which loads, weights and reactions, are applied perpendicular to its axis. It is assumed that the loads and the reactions are in a simple plane (*x*,*y* plane). Given the applied loadings, beams develop an internal shear force *V* and bending moment *M* that, in general, vary from point to point along the axis of the beam (cylinder). An expression, invoking the Bernoulli-Euler model for beams, in which the cross section plane initially perpendicular to the axis of the cylinder remains plane and perpendicular to the neutral axis during bending, can be derived (see Equation (23)). This expression provides an estimation of the axial stress along the cylinder length x- coordinate for all different scenarios considered in the experimental part of this study. At this point, it is noted that the maximum bending stress for a specific longitudinal distance is in the outer distance (exterior radius).

(23) $$\frac{\partial^{2}}{\partial x^{2}}\left(\frac{\sigma_{max}}{C}\int_{A}y^{2}dA\right)+q=0$$

where the integral represent the moment of inertia of the cross sectional area about the neutral axis, $\sigma_{max}$ is the maximum stress, *C* is the exterior radius and *q* is the distributed force. Based on the Equation (23), an estimation of the maximum stress, in MPa, along the hollow cylinder can be determined as follows: ( Nominal=12.5 ; $D_{1}=9.33$ ; $D_{2}=17.95$ ; $D_{3}=26.8$ ; $D_{4}=36.2$ ; $D_{5}=45.6$ ; $D_{6}=24.4$ ; $D_{7}=36.3$ MPa).

### 5.3. Influence of the Transducer Configuration on the Guided Wave Propagation

**Figure 4:**
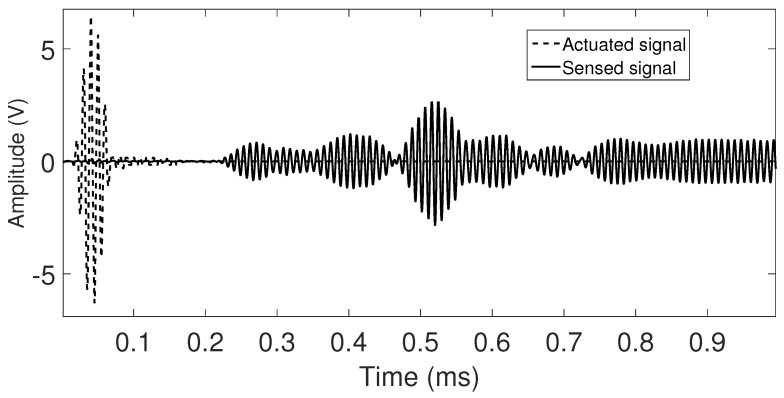
Example of actuated and captured signals in a steel pipe ϕ = 25.4 mm.

Guided waves in this study are generated by thin disks of ceramic material (PZTs) configured in radial mode. PZTs are attached to the testing specimens through an adhesive layer of cyanoacrylate, after a preparation of the surfaces and a settling time of the couplant layer. Among the suitable couplant materials cyanoacrylate has showed high repeatability with low ultrasonic impedance [27]. For the two studied specimens, the excitation pulse of the partially loading PZT attached to the specimen is a 100 KHz, five cycles Gaussian-modulated sinusoidal (See actuated signal in Figure 4). The excitation frequency is chosen to match the resonant frequency of the available PZT transducer. The contact area between PZT and the surface is around of two degrees. In consequence, the distribution of the acoustic field generated by the PZT in cylindrical waveguides diverges circumferentially, besides guided waves propagation depends on factors as mode, frequency, cylinder size, propagation and distance [28]. In addition, in the case of the studied waveguides due to a high ratio of wall thickness respect to internal radius, the cylinder contour dominates at very low frequency (wall thickness is far less than the wavelength) yielding a characteristic wave pattern, with a lot dispersion, as a result of the superposition of several guided waves, see the sensed signal shown in Figure 4.

**Figure 5:**
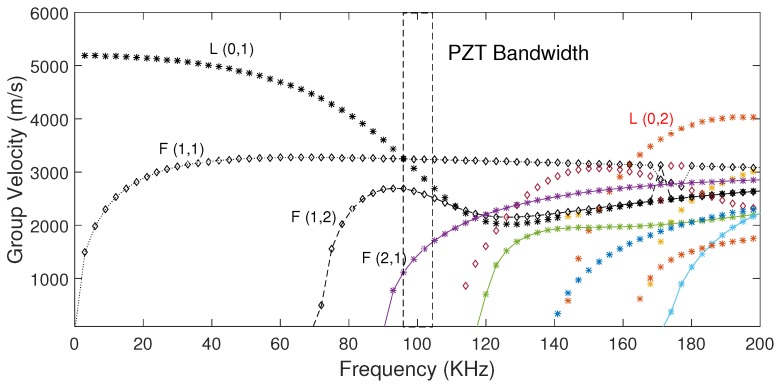
Group velocity dispersion curve for the rod.

**Figure 6:**
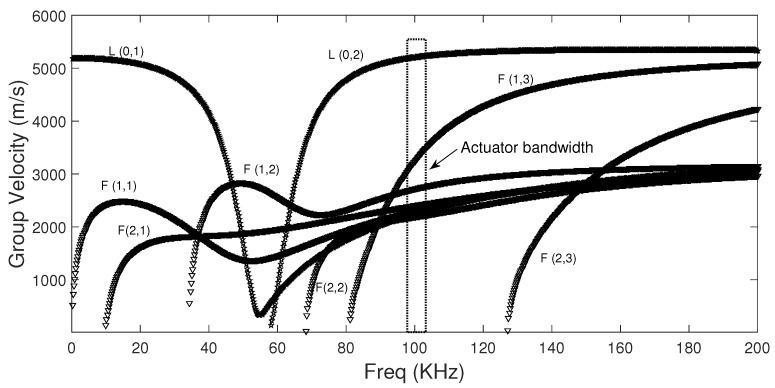
Group velocity dispersion curve for the pipe.

**Figure 7:**
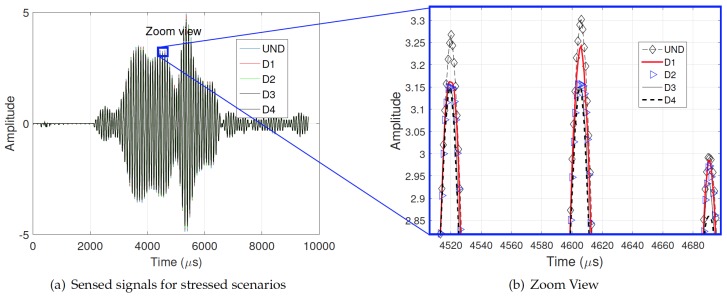
Nonuniform variations of phase shift and amplitude for different scenarios.

According to the normal mode expansion method [29], longitudinal and flexural modes ( L(0,1),F(1,1),F(1,2),F(2,1) ) should be generated by non-axisymmetric surface loading as shown in the dispersion curves depicted in Figure 5 and Figure 6 [30]. On the other hand, the contour effect generates a lot of wavepackets. Therefore, the wave pattern is rich in modes and wavepackets. Although this type of wave pattern is undesirable for TOF estimation and consequently for localization, it provides the opportunity to implement statistical tools, e.g., PCA. On the other hand, observing an example of the sensed signal for the pipe experiment, as shown in Figure 7, slight variations in the phase shift are nonlinear and almost unnoticeable changes in the amplitude of the waves for different stress scenarios (UND = 12.5, $D_{1}$ = 9.3, $D_{2}$ = 17.9, $D_{3}$ = 26.8, $D_{4}$ = 36.2 MPa). Thus, a simple conventional velocity measurement is a complex task because different velocity variations for the same stress are expected for each propagation mode, as shown in Figure 7b. This complexity and nonlinear behavior encourages the use of a statistical tool as PCA to detect any slight changes in the wave produced by different conditions of propagation.

Non-axisymmetric source loading is preferred in certain circumstances, for example when only a specific portion of the pipe is accessible, when there exists a limited number of actuators and for economic reasons. The PCA-based algorithm is programmed in Matlab, and a picoscope 2208 is used as DAQ system. A total of 100 experiments were performed and recorded for each stress scenario. The baseline model is obtained by using only 70 experiments, while 30 remaining are used for model validation. The principal components were determined by means of the baseline model, where 60 were retained for a 99.8% of data variability.

## 6. Results

Several stressed scenarios were sequentially applied on the two specimens (steel rod and hollow cylinder) during a fixed time window. Then, a DAQ and signal processing is implemented to compute the magnitude of the *Q-statistics* index for each sequential trail.

### 6.1. Rod

**Figure 8:**
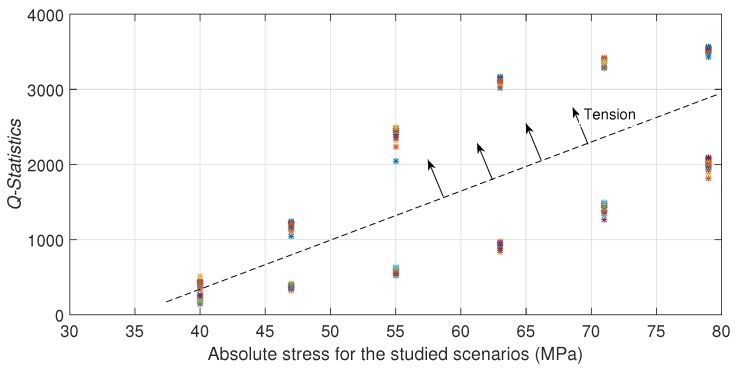
*Q-statistic* for different tension stress scenarios in the rod.

In the case of the rod, stresses were applied by using a MTS equipment. Experimental results are illustrated in Figure 8 where, it is depicted the *Q-statistic* index (Q-index) by each stress scenario. It is observed different relations between stress and Q-index: one for compression and the other for tension conditions. It can be seen also an increasing Q-index with the absolute stress in both conditions.

These results can be attributed to: (i) the different phase shift in the wavepackets, produced not only for the change in distance between the PZTs as a result of the deformation but also for the acoustoelasticity effect, and (ii) an amplitude variation of the sensed signal due to the induced voltage across the PZT terminals. This voltage is yielded by the sum of two strains: the one produced by the stress applied to the specimen which is transmitted to the PZT via an adhesive layer, and the latter produced by the dynamic perturbation (guided wave). These two reasons affect the pattern wave in different ways for each load configuration. In other words, for the same stress, the Q-index value is different for compression and tension.

This observation suggests the limitation of the proposed technique to distinguish tension and compression stresses. However, in many real applications, due to the mechanical system configuration in terms of loads, the information of the type of stress can be established a priori or considered redundant.

### 6.2. Hollow Cylinder

**Figure 9:**
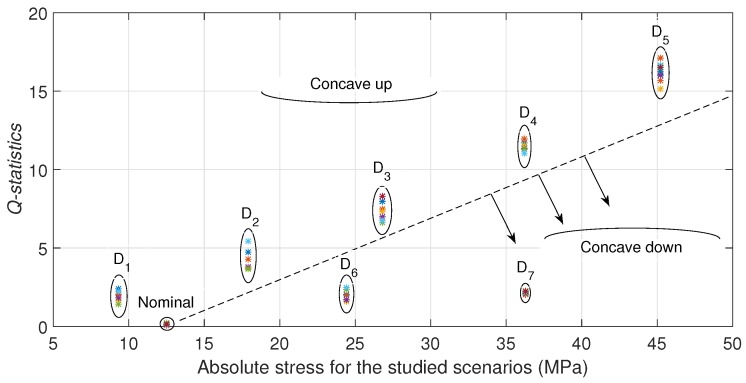
*Q-statistic* for all studied scenarios for the hollow cylinder.

**Figure 10:**
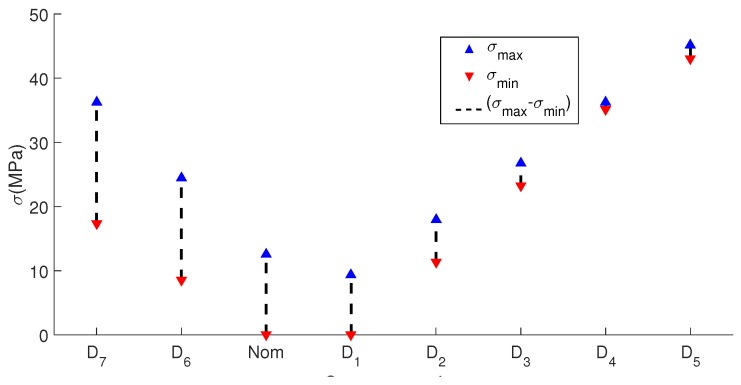
Maximum, minimum and difference of stress by each studied scenario.

**Figure 11:**
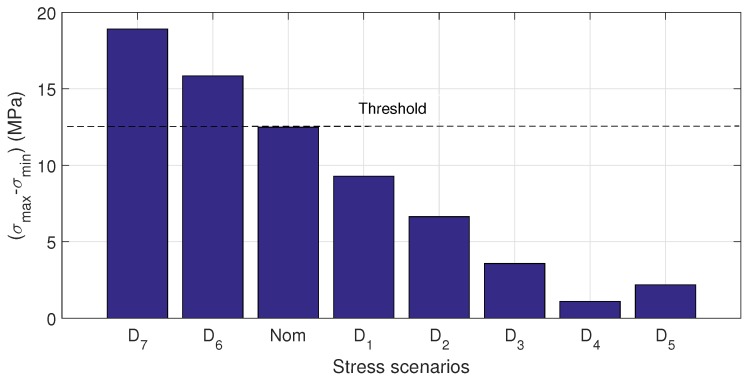
Stress difference by each studied scenario.

In the case of the hollow cylinder, each stress scenario is well defined by applying different forces in the $\frac{L}{2}$ position with a consequent normal stress generation function of the distance by bending. Experimental results are similar to the presented in the rod case. Figure 9 depicts the Q-index by each stress scenario. It is also observed different relations between stress and Q-index: for concave up and for concave down conditions. It can be seen also an increasing Q-index with the absolute stress in both conditions. It is also observed that the Q-index for the $D_{6}$ and $D_{7}$ scenarios (Concave down) present a decrease regarding the trend of the concave up scenarios. This behavior may be attributed to reasons described in the rod case and also to a wider stress range along the propagation path of the guided wave. Figure 10 and Figure 11 illustrate the difference between the maximum and the minimum stress for each scenario, e.g., $D_{4}$ exhibits $\sigma_{max}=36.22$ MPa and $\sigma_{min}=35.12$ MPa ( $\Delta_{\sigma }=1.1$ MPa), in contrast $D_{7}$ has $\sigma_{max}=36.29$ MPa and $\sigma_{min}=17.39$ MPa ( $\Delta_{\sigma }=18.9$ MPa). Clearly, a large difference means a guided wave propagation in some structure regions with much less stress than used to identify the scenario ( $\sigma_{max}$ ), so Q-index shows a consistently increment in magnitude with the stress even in a nonuniform stressed waveguide. Similarly, as shown in the rod case results, the proposed technique is not able to distinguish between concave up and concave down bending.

## 7. Discussion

In this paper, a PCA based methodology for monitoring stress conditions using PZTs is proposed and experimentally validated in two cylindrical specimens: a solid cylinder (steel rod), subject to a pure axial load, and a hollow cylinder (pipe), subject to bending. The proposed methodology is not intended to estimate stress absolute values, but instead it is able and suitable to track stress variations with respect to a nominal stress state.

The effectiveness of the proposed monitoring scheme was demonstrated to yield stress features indicators and to discriminate different stress scenarios. PCA technique, in particular the Q-index, is a good tool for detecting the presence of different stress conditions, by comparing the current index values with the nominal value via a threshold.

In a structure subjected to stress, stress variations cause changes in the arrival time of the signal due to the elongation or shortening of the path between actuator and sensor. Additionally, a change in the wave velocity is also expected due to the acoustoelasticy effect. Both mentioned effects produce a slight phase shift in the received wavepacket, which were verified by means of the experimental tests. Besides, these experiments revealed variation in amplitude for the different stress scenarios. In general, in a stress monitoring scheme, PZT-based in a pitch-catch configuration, magnitude changes in the induced voltage across the PZT terminals (i.e., amplitude of the captured signal) are yielded by the sum of the strains due to applied stress and the microstrains produced by the guided wave propagation. All these changes of the ultrasonic guided wave by propagating in a stressed medium can be easily tracked using the proposed methodology.

Specifically, the studied scenarios in the steel rod and the pipe covered the first part of the elastic region of the specimen (not damaged yet). In this region, the proposed strategy was clearly able to distinguish variations in the stress conditions with enough capability to discriminate among them. However, it does not distinguish whether the specimen is subject to tension or compression (rod case), nor the type of bending (pipe case).

Although, the proposed methodology is able to recognize different stress scenarios, the estimation of the applied stress magnitude requires further experimentation in order to determine the appropriate statistical relation. However, in the case of the rod, changes in the stress are with regard to the unstressed condition, hence it can provided a close absolute value of the actual stress once the type of load, tension or compression, is known beforehand. Therefore, the studied index constitutes a base for implementing a classifier algorithm to differentiate stress structural conditions.
